# Ogt Demonstrated Conspicuous Clinical Significance in Cancers, from Pan-Cancer to Small-Cell Lung Cancer

**DOI:** 10.1155/2022/2010341

**Published:** 2022-03-21

**Authors:** Deng Tang, Guo-Sheng Li, Ruo-Xiang Xu, Si-Yi Zhu, Jing Luo, Jin-Hua Zheng, Jun Liu, Hua-Song Lu, Mei-Hua Jin, Chong-Xi Bao, Jia Tian, Wu-Sheng Deng, Neng-Yong Zeng, Hua-Fu Zhou, Jin-Liang Kong, Gang Chen

**Affiliations:** ^1^Department of Pathology, The First Affiliated Hospital of Guangxi Medical University, Nanning 530000, China; ^2^Division of Pulmonary and Critical Care Medicine, Department of Respiratory Medicine, The First Affiliated Hospital of Guangxi Medical University, Nanning 530000, China; ^3^Department of Pathology, The Affiliated Hospital of Guilin Medical University, Guilin 541000, China; ^4^Department of Cardiothoracic Surgery, The First Affiliated Hospital of Guangxi Medical University, Nanning 530000, China; ^5^Department of Respiratory and Critical Care Medicine, The Second People's Hospital of Qinzhou, Qinzhou 535000, China

## Abstract

The clinical progression of small-cell lung cancer (SCLC) remains pessimistic. The aim of the present study was to promote the understanding of the clinical significance and mechanism of O-linked N-acetylglucosamine (GlcNAc) transferase (OGT) in SCLC. Wilcoxon tests, standardized mean difference (SMD), and Kruskal–Wallis tests were utilized to compare OGT level differences among the experimental and control groups. The univariate Cox regression analysis, Kaplan–Meier curves, and receiver operating characteristic curves were applied to determine OGT's clinical relevance in cancers. The Spearman correlation analysis and enrichment analysis were utilized to explore the underlying mechanisms of OGT in cancers. For the first time in the field, we provide an overview of OGT in 32 cancers using a large number of samples (*n* = 21,196), determining distinct OGT expression in 25 cancers and its prognosis effects in 12 cancers. Furthermore, using 950 samples from multiple sources, upregulated OGT was found in both mRNA and protein levels in SCLC (SMD = 0.93, 95% CI [0.24, 1.63]). Higher OGT levels represented a more unfavorable disease-free interval for SCLC patients (*p* < 0.001). The research also identified OGT expression as a potential marker for SCLC prediction (sensitivity = 0.79, specificity = 0.86, and AUC = 0.88). The high expression of OGT in SCLC may result from the positive regulation of two transcription factors—DEK and XRN2. We primarily investigated the underlying mechanisms of OGT in SCLC. Herein, based on the analyses from pan-cancer to SCLC, OGT demonstrated conspicuous clinical significance. OGT may be an underlying biomarker for the treatment and identification of some cancers, including SCLC.

## 1. Introduction

Lung cancer (primary bronchogenic carcinoma) has the second-highest incidence (the highest is breast cancer) and top mortality rate among cancers in the world [1]. Based on estimated worldwide data for 2020, more than 2.20 million people were newly diagnosed with lung cancer and 1.79 million individuals died of the disease [1]. According to the pathobiology features, lung cancer is classified as nonsmall-cell lung cancer (NSCLC) and small-cell lung cancer (SCLC). Compared to NSCLC, SCLC is identified as more aggressive, with a higher growth fraction and faster metastasis [2, 3], although it accounts for fewer (15%) lung cancer cases. Common therapeutic options for SCLC are lobectomy and chemotherapy combined with radiotherapy; however, the clinical progression of SCLC remains quite low, and the five-year overall survival rate of this disease has been reported at a mere 1%–5% [3–5]. Moreover, two-thirds of SCLC patients were initially diagnosed with a metastatic status [2], thus increasing the difficulty of the clinical management of the disease. Immune checkpoint inhibitors offer significant benefits to SCLC patients [2], suggesting the potential of target treatment for SCLC; however, little evidence supports the correlation of currently common biomarkers (e.g., PD-L1) with significant immunotherapy effects for SCLC [6]. In the pathogenesis of SCLC, although it is known to involve a variety of risk factors, such as tobacco smoking, the molecular mechanism has not been fully elucidated due to its complexity [7]. Thus, more effort is required to explore the potential markers and mechanisms of SCLC.

O-GlcNAcylation, a dynamic and reversible glycosylation modification, participates in a wide range of fundamental cellular processes and functions [8]. The O-linked N-acetylglucosamine (GlcNAc) transferase (OGT) protein is a glycosyltransferase encoded by the gene OGT; it enables the catalysis of a single N-acetylglucosamine (GlcNAc) molecule from uridine diphosphate N-acetylglucosamine (UDP-GlcNAc) to proteins. A variety of proteins with O-GlcNAcylation can affect the occurrence and development of malignant tumors [9]. Thus, OGT-meditated O-GlcNAcylation may provide cancer cells with an advantage for sustained growth, immune evasion, and other hallmarks in the tumor microenvironment [10]. Indeed, associations with high-OGT levels and an enhanced grade of tumor aggressiveness, heightened metastasis incidence, and poor prognosis were also identified in numerous cancers, including prostate [11], colorectal [12], ovary [13], breast [14], endometrium [15], pancreatic [16], and bladder cancers [17]. OGT has also been reported to impel the mobility and invasion of NSCLC cells by regulating O-GlcNacylation [18], demonstrating its important role in lung cancer; however, there is still a lack of research on SCLC.

The aim of the present study was to promote the understanding of the mechanism of OGT in SCLC and its clinical significance. We first performed an overview of OGT in pan-cancer and discussed its clinical value in multiple cancers. OGT expression was then explored at both the mRNA and protein levels based on in-house and multicenter SCLC samples. We also exploited the prognosis and distinction effects of OGT in SCLC and investigated the underlying molecular mechanism of the gene in the disease, contributing to a better understanding of the pathogenesis of SCLC.

## 2. Materials and Methods

**Figure 1:**
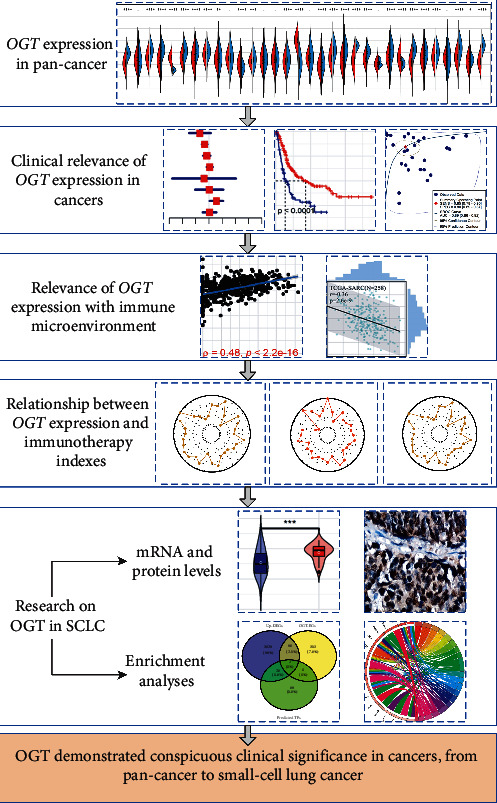
A flow chart of this study.

This study was carried out with the approval of the Ethics Committee of the First Affiliated Hospital of Guangxi Medical University and the Ethics Committee of the Affiliated Hospital of Guilin Medical University. A process flow chart of this study can be viewed in Figure 1.

### 2.1. Collection of Pan-Cancer Samples and SCLC Samples

A normalized pan-cancer cohort, containing samples from the Cancer Genome Atlas (TCGA), was obtained from the Xena database constructed by the University of California, Santa Cruz. Six types of samples in the cohort were included: “samples from solid tissue normal,” “primary solid tumor,” “primary tumor,” “normal tissue,” “primary blood-derived cancer—bone marrow,” and “primary blood-derived cancer—peripheral blood.” Noncancer tissue samples were collected from the Genotype-Tissue Expression (GTEx) database, which was combined with the TCGA cohort to explore the mRNA expression of OGT. For each type of cancer, more than three respective samples were collected for both the cancer group and the noncancer control group for further analysis. Thirty-two cancers (*n* of samples = 21,196) in the TCGA-GTEx cohort were ultimately included in the study (Table S1).

**Figure 2:**
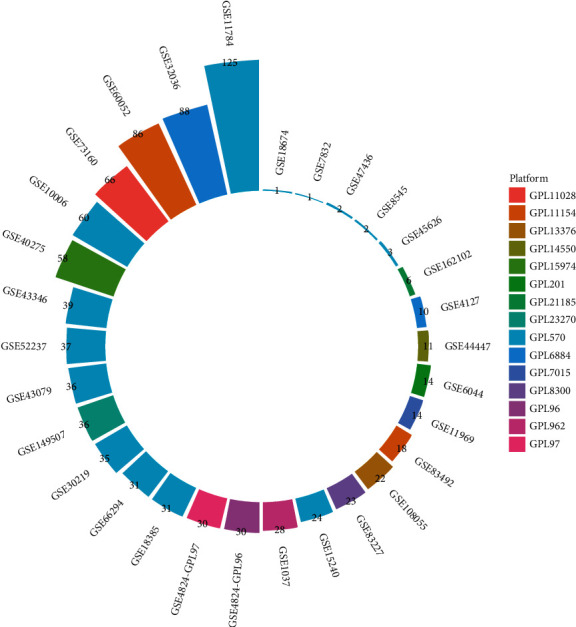
Gene Expression Omnibus cohorts included in the study and their sample number.

To analyze OGT mRNA expression and its clinical significance to SCLC (a cancer was not contained in the TCGA-GTEx cohort), cohorts from the Gene Expression Omnibus (GEO) were collected. Strategies for screening these cohorts included “(lung or bronch*∗*) and (small cell) and (mRNA or gene)”. GEO cohorts were included in this study, provided that they met the following criteria: (1) samples consisted of lung/bronchus tissues or cells of *Homo sapiens* and (2) expression profiles included mRNA levels. Cohorts were excluded if they had one of the following features: (1) contained duplicate samples from another cohort, (2) complete expression data was unavailable, and/or (3) there were only one or two samples in the combined dataset. The process of selecting the GEO cohorts is shown in Figure S1, and the included cohorts and their sample numbers are shown in Figure 2.

Collected from the First Affiliated Hospital of Guangxi Medical University and the Affiliated Hospital of Guilin Medical University, an in-house cohort with 26 SCLC samples and 29 nonSCLC samples were used to compare the differential levels of OGT protein between SCLC and nonSCLC tissues. A rabbit anti-OGT antibody, purchased from Abcam plc, was used for the immunohistochemical experiment. The experiment was performed following the manufacturer's instructions. The immunohistochemical experimental methods and protein level scoring criteria were consistent with our previous study [19].

### 2.2. Gene Expression Data Processing

The mRNA expression profiles of the TCGA-GTEx cohort and GEO cohorts were transformed by log_2_ (*x* + 1). Thirty SCLC cohorts were classified into 15 new cohorts based on the same platforms. Batch effects in merged cohorts (e.g., GPL6884, consisting of GSE32036 and GSE4127) were eliminated using the limma software package [20–22]. Finally, the mRNA expression levels in the SCLC cohorts were normalized via the limma package.

### 2.3. Mutation Landscapes of OGT in Pan-Caner

A simple nucleotide variation (SNV) dataset and a copy number variation (CNV) dataset, respectively processed by MuTect2 [23–25] and GISTIC [26–29] software, were collected from the Genomic Data Commons database. Samples in the two datasets with OGT expression equal to zero were excluded. Cancers with less than three samples were screened out. Ultimately, the SNV data of 16 cancers and the CNV data of 19 cancers were retained for further analysis. Based on the SNV dataset, the mutation landscape of OGT in pan-cancer was explored with the help of the matfools software package [22, 30, 31].

### 2.4. Clinical Associations of OGT Expression in Cancers

The relationships between OGT expression and several clinical features, including age and TNM stages, were evaluated using Kruskal–Wallis tests. OGT expression was considered independent of these clinical parameters if it was not closely related to the latter.

The relevance of OGT expression to patients' prognoses was also analyzed by Kaplan–Meier curves and univariate Cox analyses. Four clinical outcomes reflecting patients' prognoses were considered in the study: overall survival (OS), disease-specific survival (DSS), progression-free interval (PFI), and disease-free interval (DFI).

The ability of OGT expression to differentiate between cancer from noncancer samples was tested using the area under the curve (AUC) of the receiver operating characteristic curves (ROCs). The larger an AUC value is (ranging from 0 to 1), the more likely OGT expression represents conspicuous effects in identifying cancers, indicating its potential in screening for these diseases.

### 2.5. Relevance of OGT Expression with Immune Microenvironment (IME)

ESTIMATE [32–35] is a tool used to measure scores of patients based on gene expression profiles, including a stromal score (for stromal cells), an immune score (for immune cells), and an estimated score (for tumor purity). Another algorithm, TIMER [36–39], can be used to detect immune cell infiltration levels. In this study, both the ESTIMATE and TIMER algorithms were applied to explore the associations (evaluated by the Spearman correlation coefficient) between OGT expression and IME.

### 2.6. Relationship between OGT Expression and Immunotherapy Indexes

Tumor mutational burden (TMB), microsatellite instability (MSI), and homologous recombination deficiency (HRD) are considered promising indicators in immunotherapy [40–42]. Analyses of the correlation of OGT expression with the three markers were performed in this study.

### 2.7. Research on OGT in SCLC

In addition to mutation landscapes (data limited), the analyses used for pan-cancer were also applied to the research on OGT in SCLC. Moreover, both standardized mean difference (SMD) and enrichment analyses were utilized at this stage. For a coding gene, it is the protein it encodes rather than its mRNA molecule that plays a biological role. Therefore, OGT protein levels between SCLC and nonSCLC tissues were detected based on the in-house cohort of the study.

A gene with an absolute value of log_2_ (fold change) ≥1 and an SMD value >1 was considered an upregulated differently expressed gene (Up-DEGs). A gene that demonstrated a positive correlation (Pearson coefficient ≥0.3) with OGT expression in at least three cohorts was defined as an OGT-positively related gene (OGT-RG). OGT-related Up-DEGs were obtained from the intersection of Up-DEGs and OGT-RGs.

The Cistrome Data Browser [43, 44] is a database that incorporates experimental data on the chromatin immunoprecipitation sequence (ChIP-Seq) to predict transcription factors (TFs) for target genes; this method was applied to predict TFs for OGT (based on one kb base sequence upstream of OGT's transcription start site). Potential TFs likely regulating OGT expression were the intersection of predicted TFs, Up-DEGs, and OGT-RGs.

### 2.8. Other Statistical Analyses

Wilcoxon and Kruskal–Wallis tests were utilized to compare OGT level differences among the experimental and control groups (e.g., SCLC vs. nonSCLC). In the study, without any special description, *p* < 0.05 indicated that the results were statistically significant.

## 3. Results

### 3.1. The Expression of OGT and Its Mutation Landscape in Pan-Cancer

**Figure 3:**
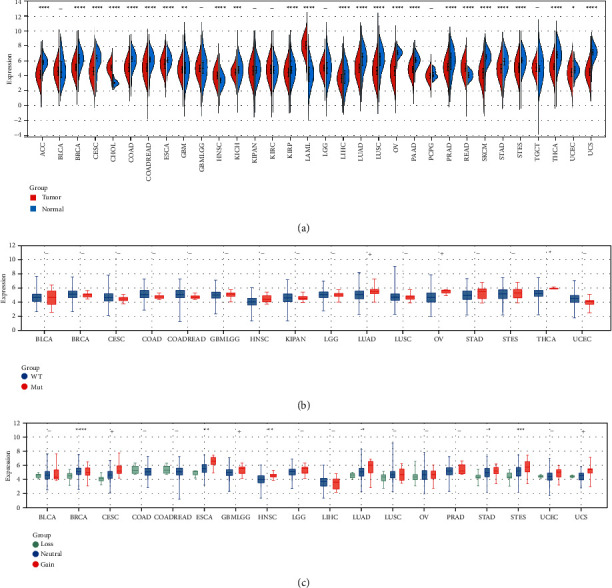
The he OGT mRNA expression and its correlation with simple nucleotide variation and copy number variation in pan-cancer (a) mRNA expression; ^*∗*^*p* value of Kruskal–Wallis tests <0.05. (b) Simple nucleotide variation. (c) Copy number variation; ^*∗*^*p* value of Wilcoxon tests <0.05.

Distinct OGT expression was observed in 25 of the 32 cancers included in this study. The upregulation of OGT expression was detected in four cancers—CHOL, HNSC, LAML, and READ. Downregulated OGT expression was determined in 21 cancers containing ACC, BRCA, CESC, COAD, COADREAD, ESCA, GBM, KICH, KIRP, LIHC, LUAD, LUSC, OV, PAAD, PRAD, SKCM, STAD, STES, THCA, UCEC, and UCS (Figure 3(a)).

All 16 cancers showed SNVs (mainly missense mutations) ranging from 0.6% to 5.1%, and the top three cancers with the highest frequency of SNV were UCEC, UCS, and LUAD (Figure S2). For the three cancers, SNVs were more likely to be detected in the high-OGT expression group than in the low-OTG expression group, and the top five genes with the highest frequency of SNV were MUC17, PIK3CA, SI, SYNE1, and PTEN (Figure S3). In THCA, upregulated OGT expression was observed in the mutant group instead of in the wild-type group (Figure 3(b)). The expression level of OGT was consistent with the trend of CNV in BRCA, ESCA, HNSC, LUAD, STAD, and STES (Figure 3(c)).

### 3.2. Clinical Relevance of OGT Expression in Cancers

**Figure 4:**
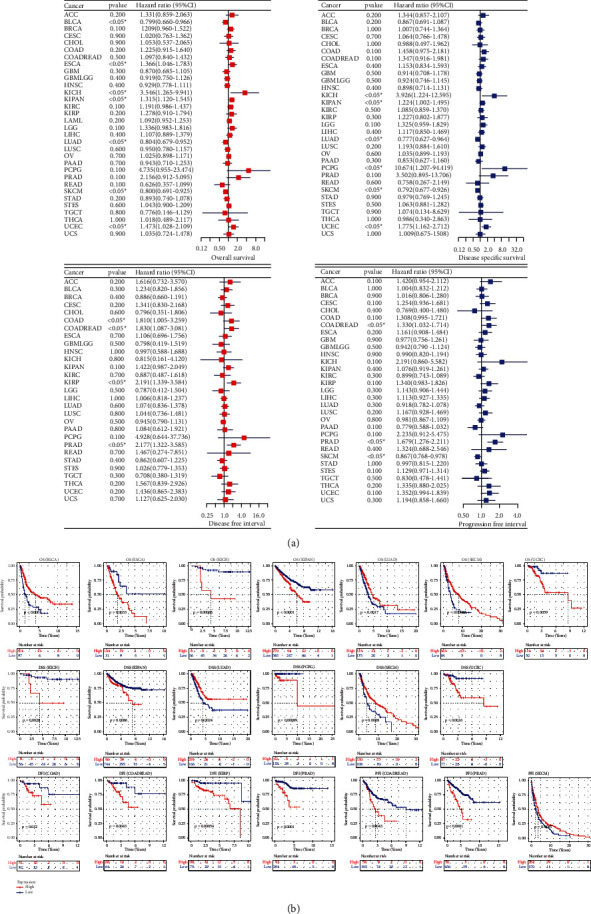
The prognosis significance of OGT expression in pan-cancer.

For the detection of OGT's prognosis significance in pan-cancer, analyses of univariate Cox and Kaplan–Meier curves were performed. No relationship of OGT expression with age and TNM stages was found in most cancers (Figure S4), suggesting OGT's independence of these features. In clinical outcomes, OGT expression plays different roles in various cancers. OGT expression is related to the favorable OS of patients with BLCA, LUAD, and SKCM, while it represented an unfavorable OS of patients with ESCA, KICH, KIPAN, and UCEC (Figure 4(a)). In addition, high-OGT expression indicated longer DSS (LUAD and SKCM) and PFI (SKCM) in some cancers, while it suggested poor DSS (KICH, KIPAN, PCPG, and UCEC), PFI (COADREAD and PRAD), and DFI (COAD, COADREAD, KIRP, and PRAD) for multiple cancers (Figure 4(a)). The prognosis significance of OGT expression in pan-cancer was also supported by the Kaplan–Meier curves (Figure 4(b)).

**Figure 5:**
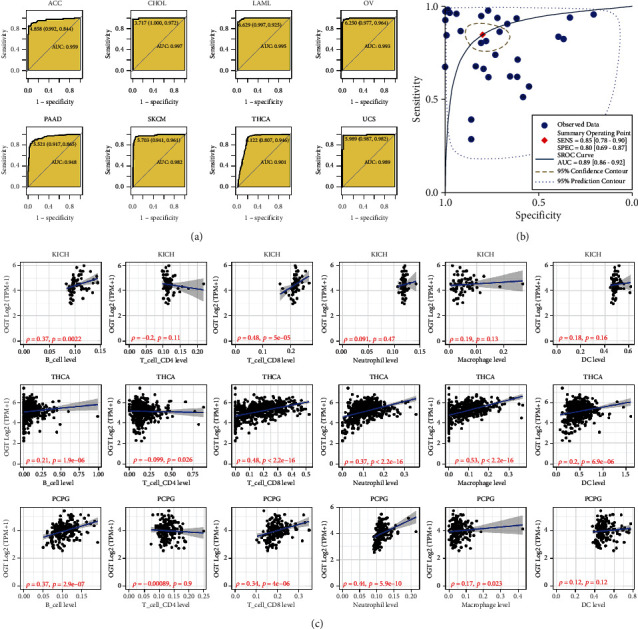
Distinguish effects of OGT expression for cancers and its relevance with immune cells infiltration. (a) Receiver operating characteristic curves. (b) Summary receiver operating characteristic curves. SENS, sensitivity; SPEC, specificity; AUC, area under the curve. (c) Associations of OGT expression and immune cells infiltration levels based on the TIMER algorithm.

In addition to prognosis significance, another clinical value—the prediction effect of OGT in pan-cancer—was explored. In 20 of the 32 cancers, the AUC values of OGT expression in differentiating cancer samples from noncancer samples were >0.7 (Figure 5(a) and Figure S5). AUC values for eight cancers (e.g., ACC) were up to at least 0.9 (Figure 5(a)), suggesting conspicuous effects of OGT expression in distinguishing cancers from noncancers. A pooled AUC (= 0.89) of the 32 cancers also supported the conclusions (Figure 5(b)).

### 3.3. Relevance of OGT Expression with IME

IME, including stromal cells and immune cells, plays an important role in tumor progression. Therefore, the association of OGT expression with IME was investigated. Through the TIMER algorithm, OGT expression was observed to have the most significant relevance with CD8 T cells in KICH, THCA, and PCPG (Spearman's correlation coefficient ≥0.34, *p* < 0.05). OGT expression was also associated with infiltration levels of B cells and neutrophils in some/all of the three cancers (Figure 5(c)).

**Figure 6:**
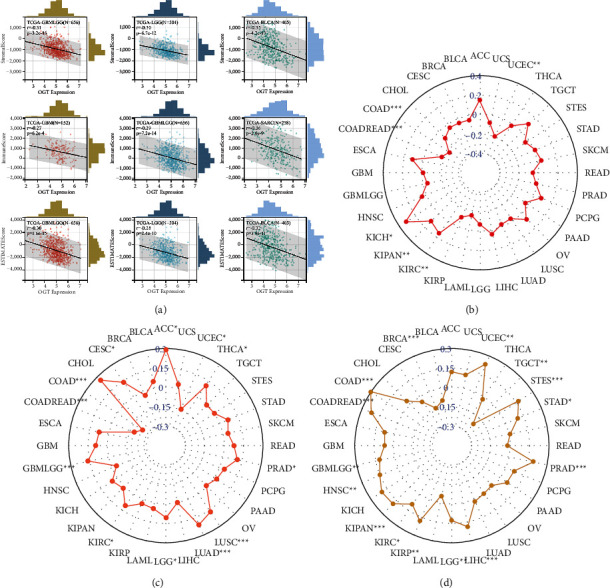
Relationship of OGT expression with the immune environment (a) and immunotherapy indexes (b)–(d). TMB, tumor mutational burden; MSI, microsatellite instability; MMR, mismatch repair; HRD, homologous recombination deficient.

Based on the ESTIMATE algorithm, negative associations of OGT expression with the stromal score, immune score, and/or estimated score were detected in most of the cancers. The most significant negative correlations between OGT expression and both the stromal score and the estimated score were observed in GBMLGG, LGG, and BLCA. The most obvious negative relation between OGT expression and the immune score was detected in GBM, GBMLGG, and SARC (Figure 6(a)).

### 3.4. Relationship between OGT Expression and Immunotherapy Indexes

The relevance of OGT expression with immunotherapy indexes was studied to determine whether OGT can be considered a potential immune treatment marker. Slight-to-moderate correlations of OGT expression with TMB, MSI, and HRD were detected in numerous cancers. A positive correlation between OGT expression and TMB was observed in KICH and that for MSI and HRD were ACC and COAD, respectively (Figures 6(b)–6(d)). A negative relationship of OGT expression with TMB was detected in COAD and that for MSI and HRD were COAD and TGCT (Figures 6(b)–6(d)).

### 3.5. Research on OGT in SCLC

The clinical progression of SCLC remains pessimistic. Efforts focusing on exploiting novel biomarkers are needed, for which we performed further research on the understanding of the clinical significance and mechanism of OGT in SCLC.

### 3.6. mRNA and Protein Levels of OGT in SCLC

**Figure 7:**
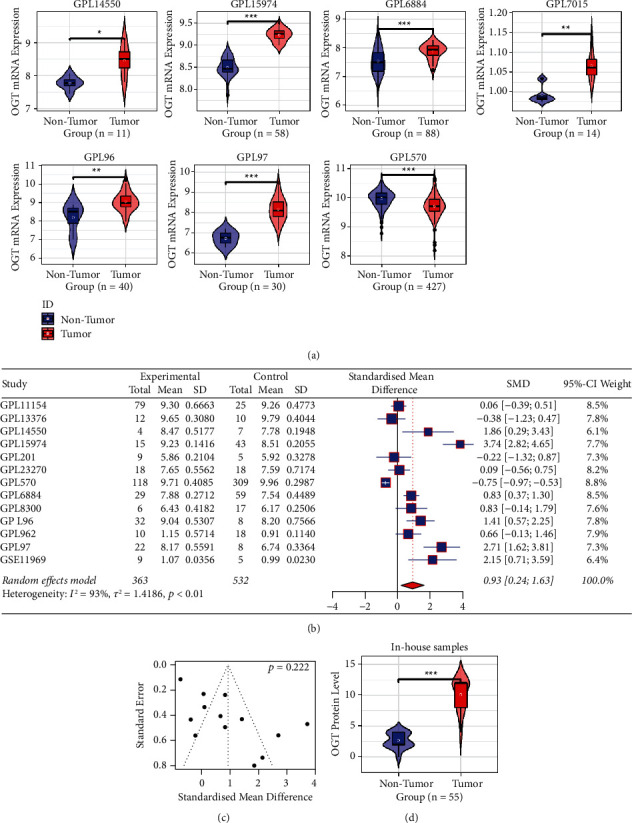
The expression of OGT in small cell lung carcinoma (SCLC). (a) Violin plots of OGT expression in SCLC. (b) A forest plot evaluating standard mean difference (SMD) of OGT expression between SCLC and nonSCLC groups. (c) A funnel plot with Begg's test for publication bias test. (d) A violin plot of OGT protein levels between SCLC and nonSCLC groups.

Among the 15 SCLC-related datasets, OGT expression was observed as upregulated (compared to nonSCLC) in six datasets, while its down-regulation was observed in one dataset (GPL570; Figure 7(a)). The other eight datasets did not indicate that the expression of OGT was statistically different between the SCLC group and the nonSCLC group (e.g., GPL11154) (Figure S6). Taken together, overexpressed OGT rather than underexpressed was observed in SCLC, which was supported by the random-effects model (SMD = 0.93, 95% CI [0.24, 1.63]; Figure 7(b)). No significant publication bias of SMD was detected through Begg's test (*p* > 0.1, Figure 7(c)).

**Figure 8:**
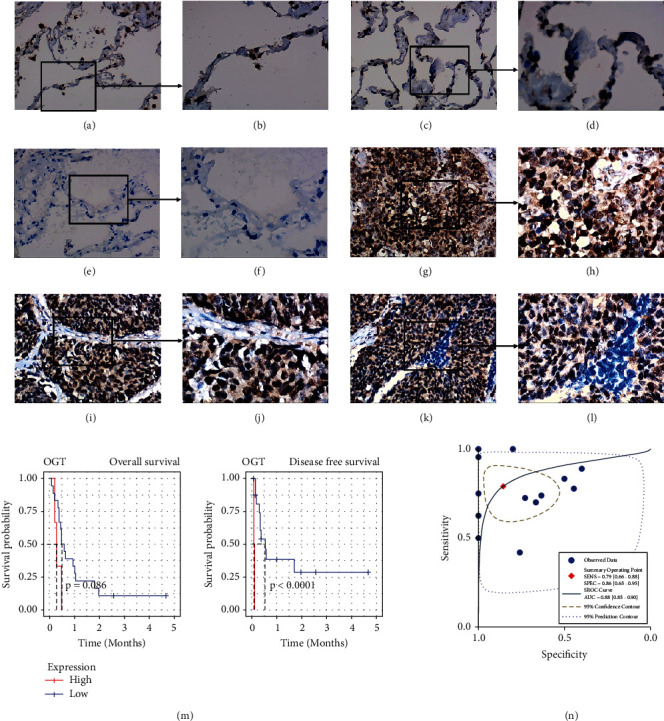
OGT protein levels and the clinical significance of OGT expression in small cell lung carcinoma (SCLC). (a)–(l) The protein levels of OGT in nonSCLC (a–f) and SCLC (g–l) tissues under the microscope by in-house tissue microarrays. The left figure of each two combined figures is 200x, and the right figure is 400x. (m) Kaplan–Meier curves of survival between high- and low-OGT expression groups. (n) A summary receiver operating characteristic curve for identifying small cell lung carcinoma based on OGT expression. SENS, sensitivity; SPEC, specificity; AUC, area under the curve.

To confirm OGT expression in SCLC, we investigated an in-house immunohistochemical experiment. As a result, compared to nonSCLC tissues, increased OGT protein levels were found in SCLC tissues (Figure 7(d)), consistent with its mRNA expression. Under the microscope, positive OGT protein levels were observed in the SCLC tissues rather than in their control tissues (Figures 8(a)–8(l)).

### 3.7. Clinical Correlation of OGT Expression in SCLC

No significant differences in OGT expression between various ages and TNM stages were detected (Figure S7). Upregulated OGT expression tended to be associated with poor OS (*p* = 0.086) and DFI (*p* < 0.001; Figure 8(m)). OGT expression enabled the conspicuous differentiation of SCLC samples and nonSCLC samples (sensitivity = 0.79, specificity = 0.86, and AUC = 0.88; Figure 8(n)), similar to the results of the pan-cancer analysis.

### 3.8. Potential Mechanism of High-OGT Expression in SCLC

**Figure 9:**
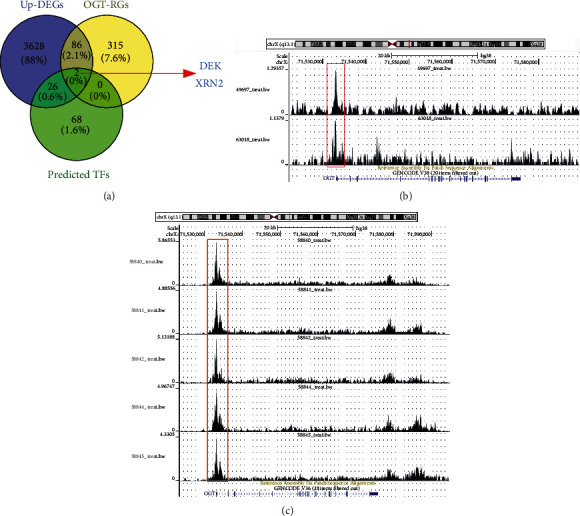
Identification of potential transcription factors regulating OGT expression. For the two transcription factors, there existed binding sites with the potential promoter region of OGT.

Via the calculation, 3742 Up-DEGs, 403 OGT-RGs, and 96 predicted TFs were identified. Two TFs (DEK and XRN2) selected from the intersection of these genes (Figure 9(a)) were considered potential TFs regulating OGT expression. This conclusion was also supported by the ChIP-Seq peaks of DEK and the XRN2 upstream of OGT's transcription start site (Figure 9(b)–9(c)).

### 3.9. Enrichment Analyses and IME of OGT in Cancer

**Figure 10:**
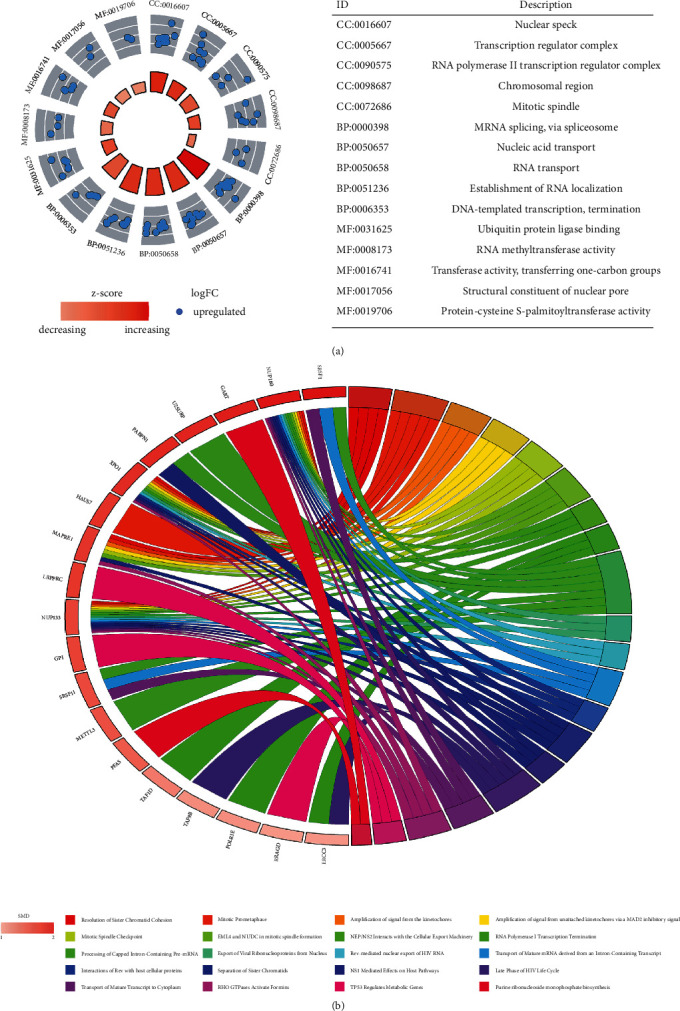
Gene Ontology terms and signaling pathways of OGT-related upregulated expression genes. CC: cellular component; BP: biological process; MF: molecular function.

Eighty-nine OGT-related Up-DEGs were screened for enrichment analyses. In GO terms, these genes were involved in mitotic and transcription regulators (cell components), participated in mRNA splicing and nucleic acid transport (biological processes), and linked with ubiquitin protein-ligase binding and RNA methyltransferase activity (molecular functions; Figure 10(a)). In addition, OGT-related Up-DEGs clustered in 20 Reactome signaling pathways, such as “Resolution of Sister Chromatid Cohesion” and “Mitotic Prometaphase” (Figure 10(b)).

No statistical difference was detected for OGT expression and IME; however, a trend was observed in SCLC that OGT expression was negatively related to the stromal score and immune score (Figure S8(a)). High-OGT expression tended to negatively relate to infiltration levels of CD8 T cells and M0 macrophages and was positively associated with resting CD4 memory T cells (Figure S8(b)).

## 4. Discussion

In our study, we investigated the expression, clinical relevance, and underlying mechanisms of OGT in pan-cancer, including SCLC. For the first time in the field, we have provided an overview of OGT in 32 cancers using a large number of samples (*n* = 21,196), determining distinct OGT expression in 25 cancers and its prognosis effects in 12 cancers. Furthermore, by analyzing 950 samples from multiple sources, we observed upregulated OGT in both mRNA and protein levels in SCLC. Higher OGT levels represented a more unfavorable prognosis for SCLC patients. The research also identified OGT expression as a potential marker for SCLC prediction. The high expression of OGT in SCLC may result from the positive regulation of two TFs—DEK and XRN2. We also primarily investigated the underlying mechanisms of OGT in SCLC. Based on analyses from pan-cancer to SCLC, OGT demonstrated conspicuous clinical significance.

Aberrant OGT expression was identified and demonstrated significant associations in multiple cancers. Zhou et al. [45] identified the downregulation of OGT and its low expression status, affecting cisplatin resistance in ovarian cancer. Jin et al. [17] determined the upregulation of OGT and its oncogenic role in bladder cancer. Wu et al. [46] initially demonstrated abnormal OGT expression in various cancers by exploring 8,948 samples, but they did not investigate the clinical significance of OGT in cancers. Furthermore, our study not only distinguished OGT expression in numerous (25/32) cancers by using large samples (*n* = 21,196) but also revealed its significant association with the prognosis of patients in 12 cancers—BLCA, COAD, COADREAD, ESCA, LUAD, KICH, KIRP, KIPAN, PCPG, PRAD, SKCM, and UCEC. To the best of our knowledge, except for BLCA [47], ESCA [48], LUAD [49], and PRAD [45], the prognosis effects of OGT in the remaining eight cancers are newly reported. OGT was previously determined as an underlying marker for BLCA prediction [50], as positive OGT expression was detected in half of the patients' urine. In our study, conspicuous effects of OGT expression in distinguishing cancers from noncancers were found in eight cancers (ACC, CHOL, LAML, OV, PAAD, SKCM, THCA, and UCS), indicating its potential in cancer prediction. Thus, distinct OGT expression may serve as an essential marker in the clinical management (e.g., targeted therapy) of some cancers.

The mechanisms of OGT in various cancers may be varied and complex. O-GlcNAcylation has been reported to play important roles in immune cell activation, thus participating in immune responses [51]. Our research revealed the positive relevance of OGT expression with the filtration levels of some immune cells (particularly the CD8 T cell, a well-known anticancer cell [52]) in some cancers (e.g., THCA), and we also found a negative association between OGT expression and immune-related scores in several cancers (e.g., GBMLGG). To some extent, these findings indicate a correlation between OGT expression and immune responses; however, the mechanisms shown in various cancers were diverse and require further study. In addition, the study also showed correlations of OGT expression with TMB, MSI, and HRD (all were biomarkers in immunotherapy [40–42]), initially implying its potential in immunotherapy, which was verified by further research.

Diverse (increased) expression levels of OGT showed clinical associations in SCLC, similar to the results for pan-cancer. Although OGT expression was considered an independent risk factor for the prognostic of LUAD (one of the NSCLCs) [49], as far as we know, little is understood about OGT's roles in SCLC. In our study, upregulated OGT mRNA and protein levels were primarily determined using multicenter data and in-house samples, respectively. The high expression of OGT was correlated to unfavorable DFI of SCLC, suggesting its risk role in the prognosis of patients with the disease. Similar to a series of cancers (e.g., ACC), OGT made it feasible to distinguish SCLC from nonSCLC, implying its prediction effects for SCLC. Therefore, OGT demonstrated underlying clinical value in SCLC, and it may be a marker for the future treatment of SCLC.

OGT may also play a role in SCLC through its typical functions. The OGT protein catalyzes a single O-GlcNAcylation (GlcNAc) molecule from uridine diphosphate N-acetylglucosamine to proteins, thus upregulating O-GlcNAcylation levels. Increasing OGT-meditated O-GlcNAcylation levels could affect the occurrence and progression of cancers [9, 10], including lung cancer. For example, Ge et al. [18] demonstrated O-GlcNacylation's effects in enhancing mobility and invasion by stimulating IL-6/STAT3 signaling in NSCLC. In our study, for the first time, we revealed that TFs DEK and XRN2 may regulate OGT expression in SCLC in the following ways: (1) both TFs were Up-DEGs in SCLC, similar to OGT; (2) conspicuous and positive expression relationships of DEK and XRN2 with OGT were detected in SCLC; and (3) ChIP-Seq binding peaks of the two TFs were observed in the potential promoter region of OGT. Previously, O-GlcNAcylation was considered to participate in multiple cellular processes, such as transcription, signal transduction, and chromatin remodeling [53]. As shown by our research, based on OGT-related Up-DEGs, OGT may preserve its roles in SCLC by involving mitotic and transcription regulators (cell components), participating in mRNA splicing and nucleic acid transport (biological processes), and linking with ubiquitin protein ligase binding and RNA methyltransferase activity (molecular functions), which contributes to signaling pathways, such as “Resolution of Sister Chromatid Cohesion” and “Mitotic Prometaphase.” Such results suggest that OGT may affect the development of SCLC via its classical functions, which requires further verification.

There were several limitations of the research. Initially, we failed to collect enough samples to verify the mRNA and protein levels of OGT in cancers of the pan-cancer analysis and the relevance of OGT with the prognosis. Adequate body fluid samples were required to verify OGT's ability to distinguish cancerous from noncancerous samples. Future *in vivo* and *in vitro* investigations are needed to exploit the molecular mechanism of OGT expression in SCLC.

## 5. Conclusions

Collectively, we determined the different OGT expressions and their significant clinical values in various cancers. OGT may be an underlying biomarker for the treatment and identification of some cancers, including SCLC.

## Data Availability

The data that support the findings of pan-cancer analyses are available in public databases with serial number for each dataset and the databases containing Xena database at https://xena.ucsc.edu/, Sanger Box database at http://vip.sangerbox.com/, GTEx Portal at https://gtexportal.org/home/, Depmap Portal at https://depmap.org/portal/download/, Gene Expression Omnibus at https://www.ncbi.nlm.nih.gov/gds/, and the Cancer Genome Atlas at https://www.cancer.gov/about-nci/organization/ccg/research/structural-genomics/tcga. Data on in-house tissue samples used during the current study are available from the corresponding author on reasonable request.

## Supplementary Materials

Supplementary Materials Table S1. Thirty-two cancers in the TCGA-GTEx cohort were eventually included in the study. Figure S1. The processes of selecting GEO cohorts. Figure S2. The frequency of SNVs of OGT in pan-cancer. Figure S3. SNVs of the high-OGT expression group and the low-OTG expression group. Figure S4. The relationship of OGT expression with clinical features in cancers. Figure S5. The prediction effect of OGT in pan-cancer. Figure S6. The eight datasets did not indicate that the expression of OGT was statistically different between the SCLC group and the nonSCLC group. Figure S7. No significant differences in clinical features were detected between the SCLC and nonSCLC groups. Figure S8. No statistical difference was detected in OGT expression and IME.
