# Supplementary material for: Ogt Demonstrated Conspicuous Clinical Significance in Cancers, from Pan-Cancer to Small-Cell Lung Cancer
Source: J Oncol. 2022 Mar 21;2022:2010341. doi: 10.1155/2022/2010341 (PMC8959957; doi:10.1155/2022/2010341)
Supplement: Supplementary Materials — Table S1. Thirty-two cancers in the TCGA-GTEx cohort were eventually included in the study. Figure S1. The processes of selecting GEO cohorts. Figure S2. The frequency of SNVs of OGT in pan-cancer. Figure S3. SNVs of the high-OGT expression group and the low-OTG expression group. Figure S4. The relationship of OGT expression with clinical features in cancers. Figure S5. The prediction effect of OGT in pan-cancer. Figure S6. The eight datasets did not indicate that the expression of OGT was statistically different between the SCLC group and the nonSCLC group. Figure S7. No significant differences in clinical features were detected between the SCLC and nonSCLC groups. Figure S8. No statistical difference was detected in OGT expression and IME. [file 2010341.f1.zip › 2010341.f1/Table S1 (1).docx]

**Table S1.** Cancers (from the pan-cancer cohort) included in the study.

| Cancers | Abbreviation | *N* ^a^ of cancer group | *N* of non-cancer group |
| --- | --- | --- | --- |
| Adrenocortical carcinoma | ACC | 77 | 127 |
| Bladder urothelial carcinoma | BLCA | 407 | 28 |
| Breast invasive carcinoma | BRCA | 1092 | 292 |
| Cervical squamous cell carcinoma and endocervical adenocarcinoma | CESC | 304 | 13 |
| Cholangiocarcinoma | CHOL | 36 | 9 |
| Colon adenocarcinoma | COAD | 288 | 345 |
| Colon adenocarcinoma/rectum adenocarcinoma esophageal carcinoma | COADREAD | 380 | 355 |
| Esophageal carcinoma | ESCA | 181 | 664 |
| Glioblastoma multiforme | GBM | 153 | 1151 |
| Glioma | GBMLGG | 662 | 1151 |
| Head and neck squamous cell carcinoma | HNSC | 518 | 44 |
| Kidney chromophobe | KICH | 66 | 156 |
| Pan-kidney cohort (KICH+KIRC+KIRP) | KIPAN | 884 | 156 |
| Kidney renal clear cell carcinoma | KIRC | 530 | 156 |
| Kidney renal papillary cell carcinoma | KIRP | 288 | 156 |
| Acute myeloid leukemia | LAML | 173 | 337 |
| Brain lower grade glioma | LGG | 509 | 1151 |
| Liver hepatocellular carcinoma | LIHC | 369 | 160 |
| Lung adenocarcinoma | LUAD | 513 | 396 |
| Lung squamous cell carcinoma | LUSC | 498 | 396 |
| Ovarian serous cystadenocarcinoma | OV | 418 | 88 |
| Pancreatic adenocarcinoma | PAAD | 178 | 169 |
| Pheochromocytoma and paraganglioma | PCPG | 177 | 3 |
| Prostate adenocarcinoma | PRAD | 495 | 152 |
| Rectum adenocarcinoma | READ | 92 | 10 |
| Stomach adenocarcinoma | STAD | 414 | 209 |
| Skin cutaneous melanoma | SKCM | 102 | 558 |
| Stomach and esophageal carcinoma | STES | 595 | 873 |
| Testicular germ cell tumors | TGCT | 148 | 165 |
| Thyroid carcinoma | THCA | 504 | 337 |
| Uterine corpus endometrial carcinoma | UCEC | 180 | 23 |
| Uterine carcinosarcoma | UCS | 57 | 78 |

Notes: ^a^, number.
