# Supplementary figures and images for: Ogt Demonstrated Conspicuous Clinical Significance in Cancers, from Pan-Cancer to Small-Cell Lung Cancer
Source: J Oncol. 2022 Mar 21;2022:2010341. doi: 10.1155/2022/2010341 (PMC8959957; doi:10.1155/2022/2010341)

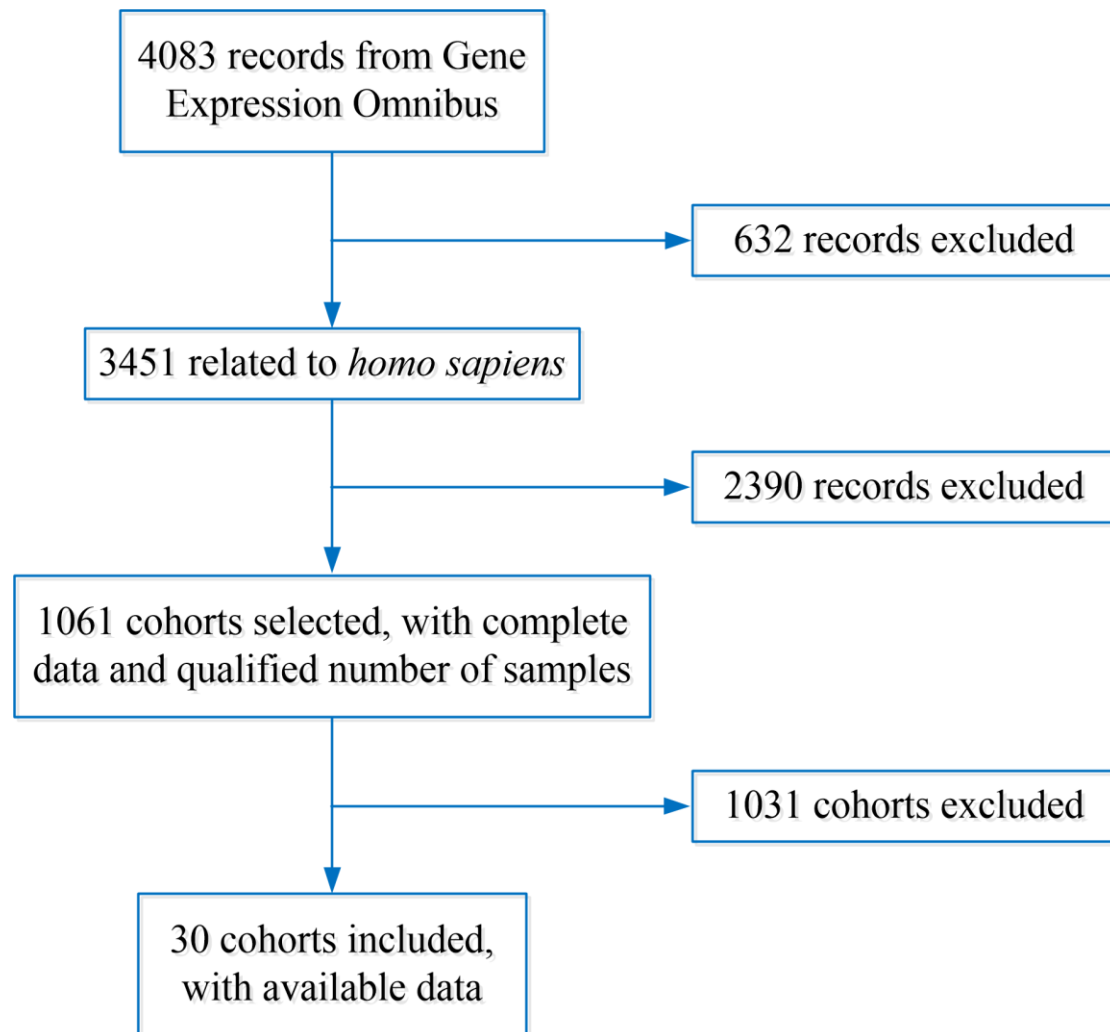

Supplement: Supplementary Materials — Table S1. Thirty-two cancers in the TCGA-GTEx cohort were eventually included in the study. Figure S1. The processes of selecting GEO cohorts. Figure S2. The frequency of SNVs of OGT in pan-cancer. Figure S3. SNVs of the high-OGT expression group and the low-OTG expression group. Figure S4. The relationship of OGT expression with clinical features in cancers. Figure S5. The prediction effect of OGT in pan-cancer. Figure S6. The eight datasets did not indicate that the expression of OGT was statistically different between the SCLC group and the nonSCLC group. Figure S7. No significant differences in clinical features were detected between the SCLC and nonSCLC groups. Figure S8. No statistical difference was detected in OGT expression and IME. [file 2010341.f1.zip › 2010341.f1/Figure S1.pdf]

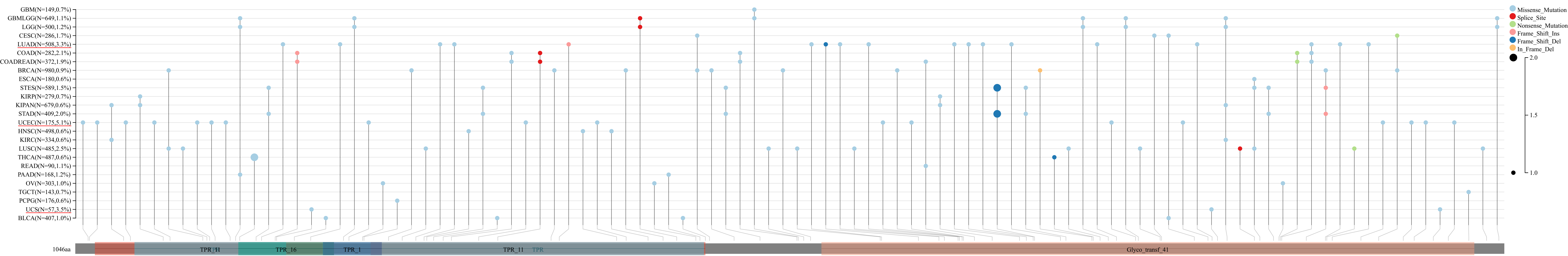

Supplement: Supplementary Materials — Table S1. Thirty-two cancers in the TCGA-GTEx cohort were eventually included in the study. Figure S1. The processes of selecting GEO cohorts. Figure S2. The frequency of SNVs of OGT in pan-cancer. Figure S3. SNVs of the high-OGT expression group and the low-OTG expression group. Figure S4. The relationship of OGT expression with clinical features in cancers. Figure S5. The prediction effect of OGT in pan-cancer. Figure S6. The eight datasets did not indicate that the expression of OGT was statistically different between the SCLC group and the nonSCLC group. Figure S7. No significant differences in clinical features were detected between the SCLC and nonSCLC groups. Figure S8. No statistical difference was detected in OGT expression and IME. [file 2010341.f1.zip › 2010341.f1/Figure S2.pdf]

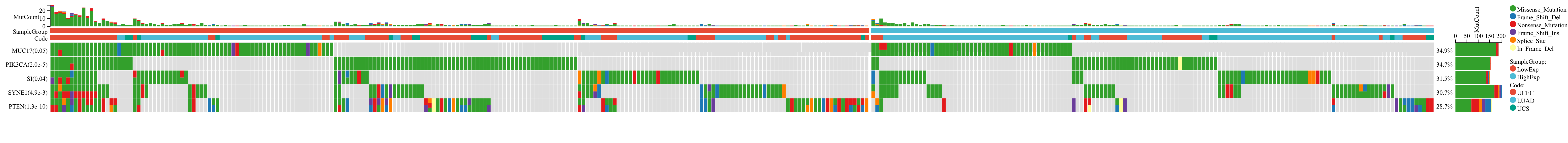

Supplement: Supplementary Materials — Table S1. Thirty-two cancers in the TCGA-GTEx cohort were eventually included in the study. Figure S1. The processes of selecting GEO cohorts. Figure S2. The frequency of SNVs of OGT in pan-cancer. Figure S3. SNVs of the high-OGT expression group and the low-OTG expression group. Figure S4. The relationship of OGT expression with clinical features in cancers. Figure S5. The prediction effect of OGT in pan-cancer. Figure S6. The eight datasets did not indicate that the expression of OGT was statistically different between the SCLC group and the nonSCLC group. Figure S7. No significant differences in clinical features were detected between the SCLC and nonSCLC groups. Figure S8. No statistical difference was detected in OGT expression and IME. [file 2010341.f1.zip › 2010341.f1/Figure S3.pdf]

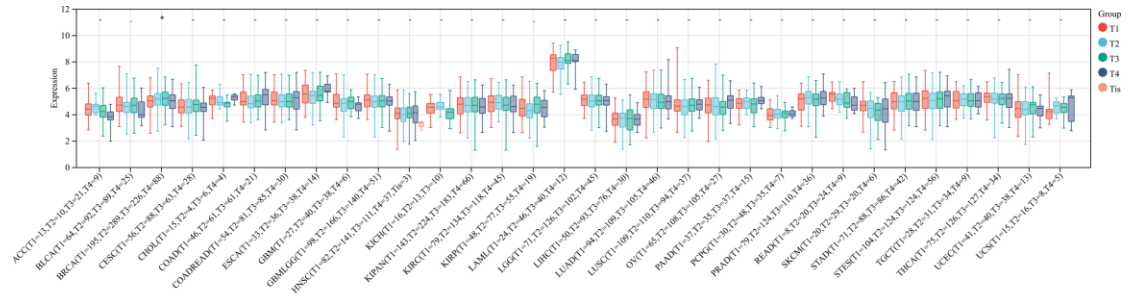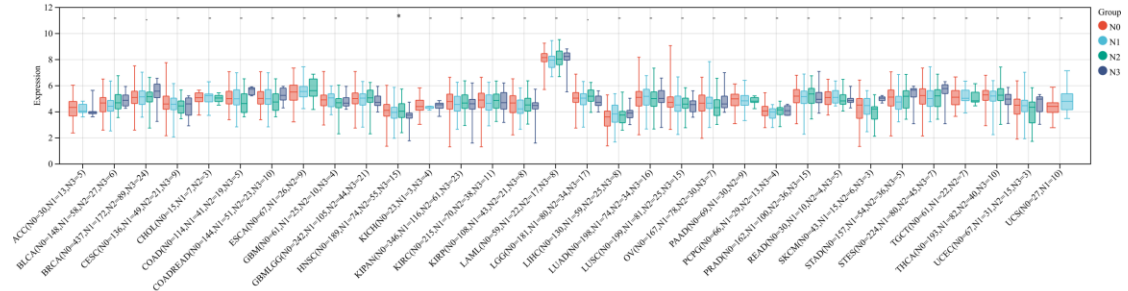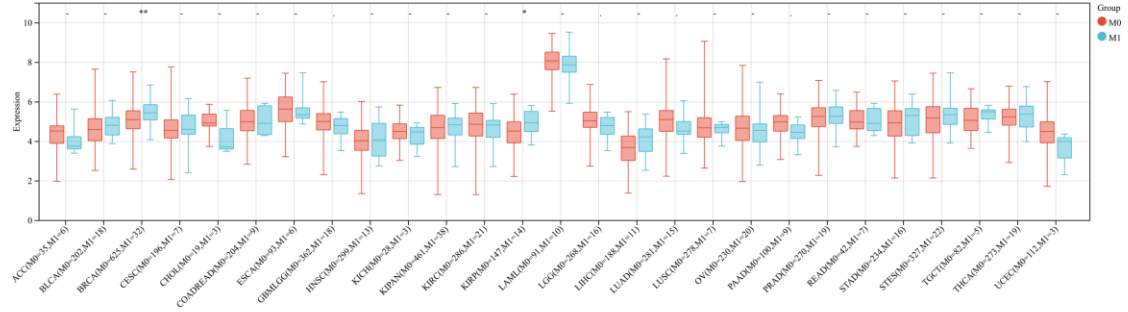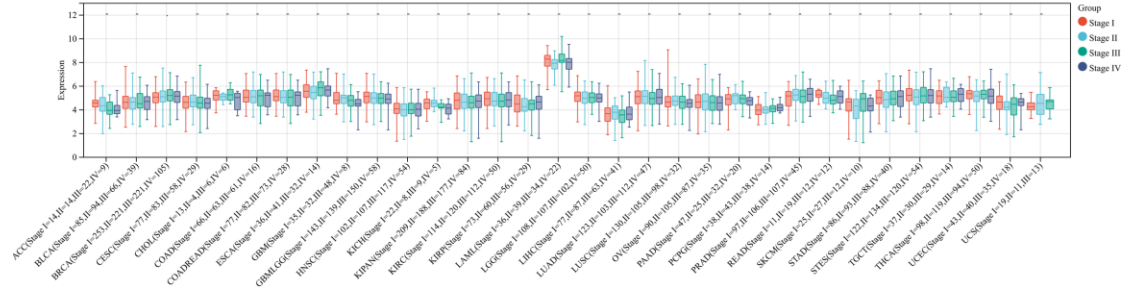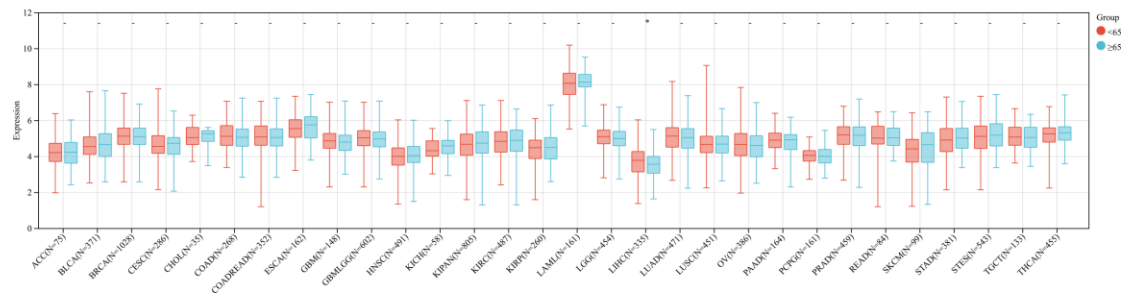

Supplement: Supplementary Materials — Table S1. Thirty-two cancers in the TCGA-GTEx cohort were eventually included in the study. Figure S1. The processes of selecting GEO cohorts. Figure S2. The frequency of SNVs of OGT in pan-cancer. Figure S3. SNVs of the high-OGT expression group and the low-OTG expression group. Figure S4. The relationship of OGT expression with clinical features in cancers. Figure S5. The prediction effect of OGT in pan-cancer. Figure S6. The eight datasets did not indicate that the expression of OGT was statistically different between the SCLC group and the nonSCLC group. Figure S7. No significant differences in clinical features were detected between the SCLC and nonSCLC groups. Figure S8. No statistical difference was detected in OGT expression and IME. [file 2010341.f1.zip › 2010341.f1/Figure S4.pdf]

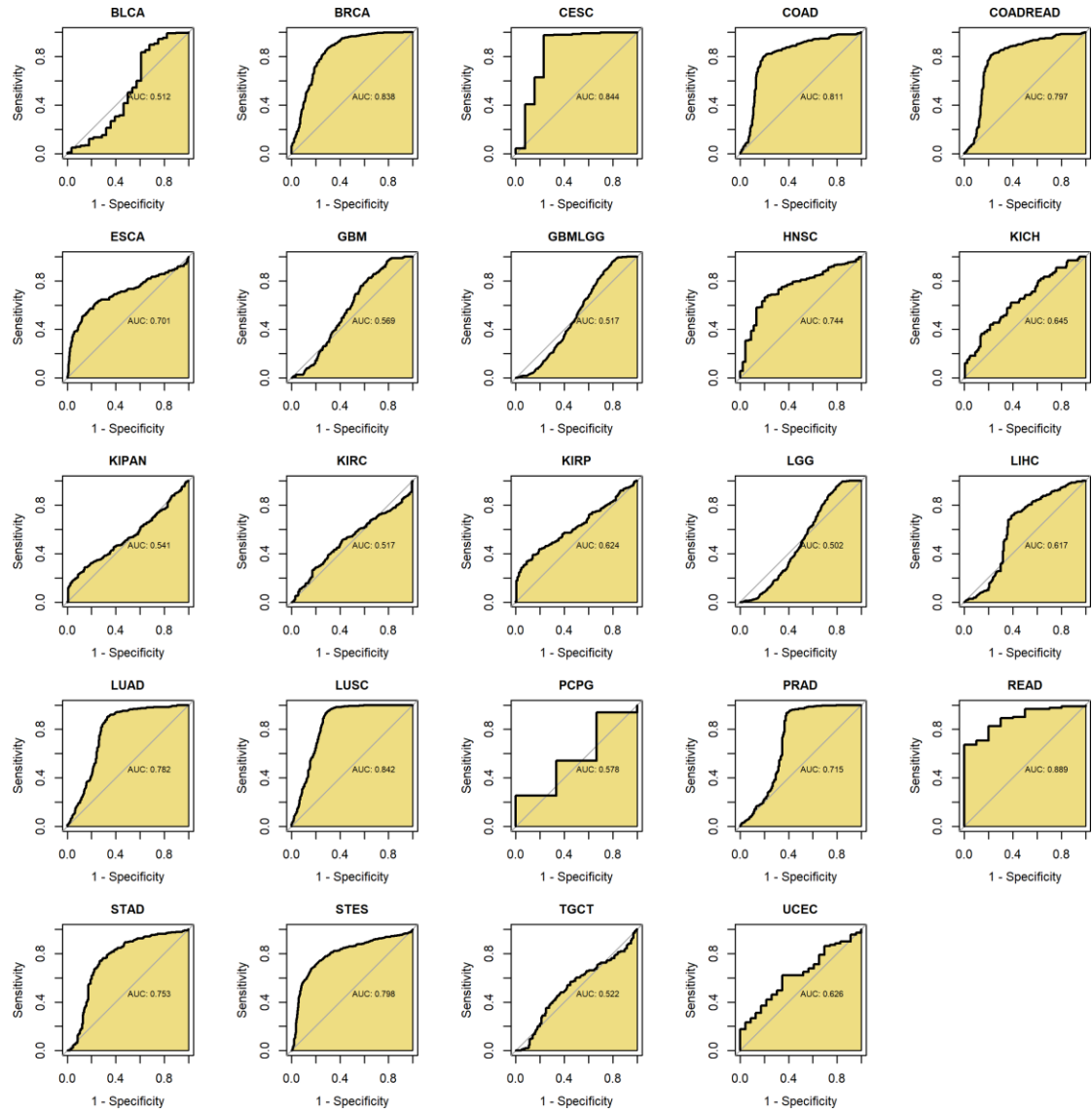

Supplement: Supplementary Materials — Table S1. Thirty-two cancers in the TCGA-GTEx cohort were eventually included in the study. Figure S1. The processes of selecting GEO cohorts. Figure S2. The frequency of SNVs of OGT in pan-cancer. Figure S3. SNVs of the high-OGT expression group and the low-OTG expression group. Figure S4. The relationship of OGT expression with clinical features in cancers. Figure S5. The prediction effect of OGT in pan-cancer. Figure S6. The eight datasets did not indicate that the expression of OGT was statistically different between the SCLC group and the nonSCLC group. Figure S7. No significant differences in clinical features were detected between the SCLC and nonSCLC groups. Figure S8. No statistical difference was detected in OGT expression and IME. [file 2010341.f1.zip › 2010341.f1/Figure S5.pdf]

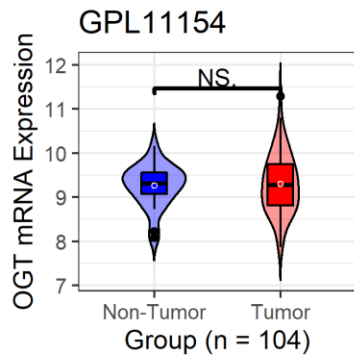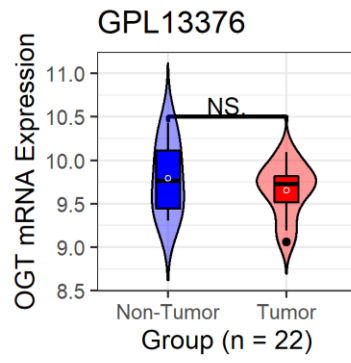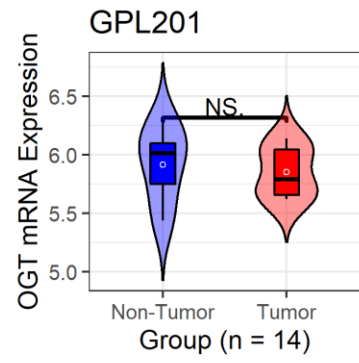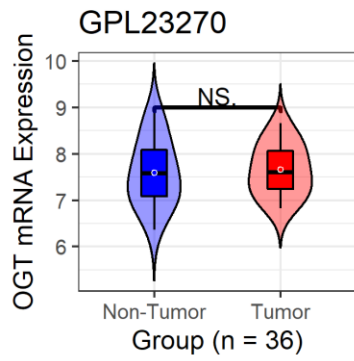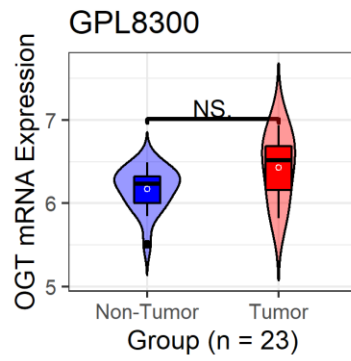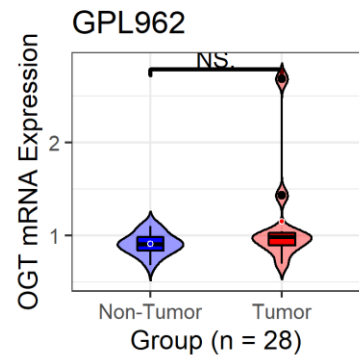

Supplement: Supplementary Materials — Table S1. Thirty-two cancers in the TCGA-GTEx cohort were eventually included in the study. Figure S1. The processes of selecting GEO cohorts. Figure S2. The frequency of SNVs of OGT in pan-cancer. Figure S3. SNVs of the high-OGT expression group and the low-OTG expression group. Figure S4. The relationship of OGT expression with clinical features in cancers. Figure S5. The prediction effect of OGT in pan-cancer. Figure S6. The eight datasets did not indicate that the expression of OGT was statistically different between the SCLC group and the nonSCLC group. Figure S7. No significant differences in clinical features were detected between the SCLC and nonSCLC groups. Figure S8. No statistical difference was detected in OGT expression and IME. [file 2010341.f1.zip › 2010341.f1/Figure S6.pdf]

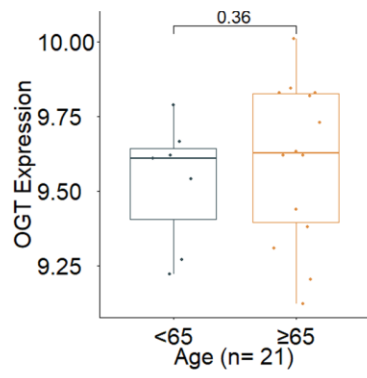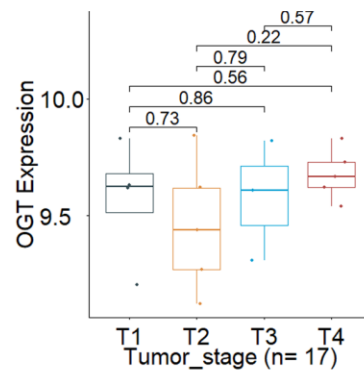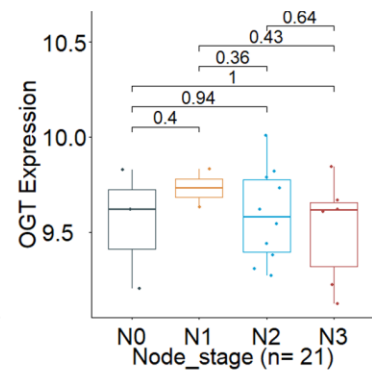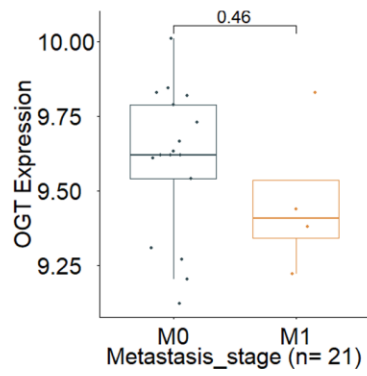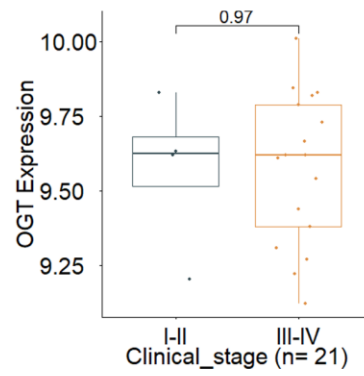

Supplement: Supplementary Materials — Table S1. Thirty-two cancers in the TCGA-GTEx cohort were eventually included in the study. Figure S1. The processes of selecting GEO cohorts. Figure S2. The frequency of SNVs of OGT in pan-cancer. Figure S3. SNVs of the high-OGT expression group and the low-OTG expression group. Figure S4. The relationship of OGT expression with clinical features in cancers. Figure S5. The prediction effect of OGT in pan-cancer. Figure S6. The eight datasets did not indicate that the expression of OGT was statistically different between the SCLC group and the nonSCLC group. Figure S7. No significant differences in clinical features were detected between the SCLC and nonSCLC groups. Figure S8. No statistical difference was detected in OGT expression and IME. [file 2010341.f1.zip › 2010341.f1/Figure S7.pdf]

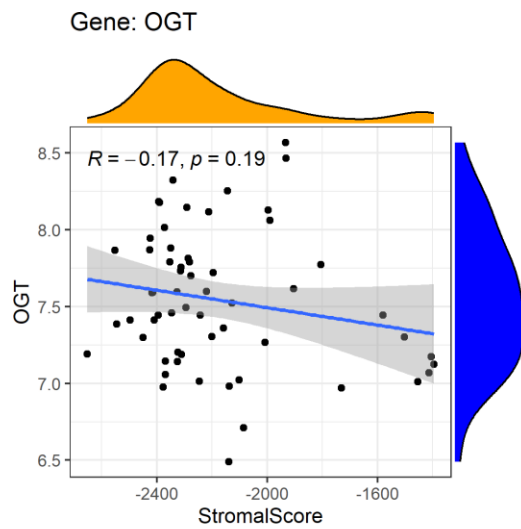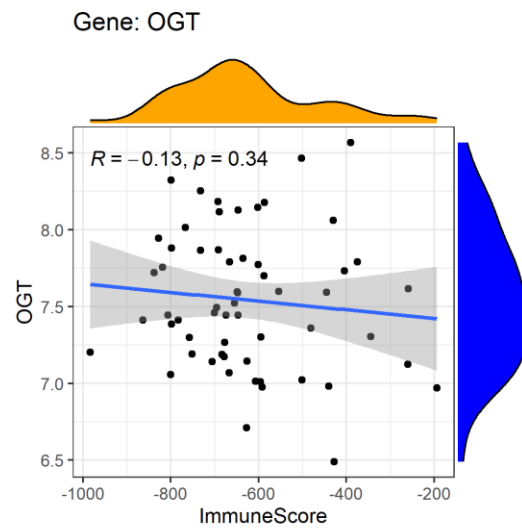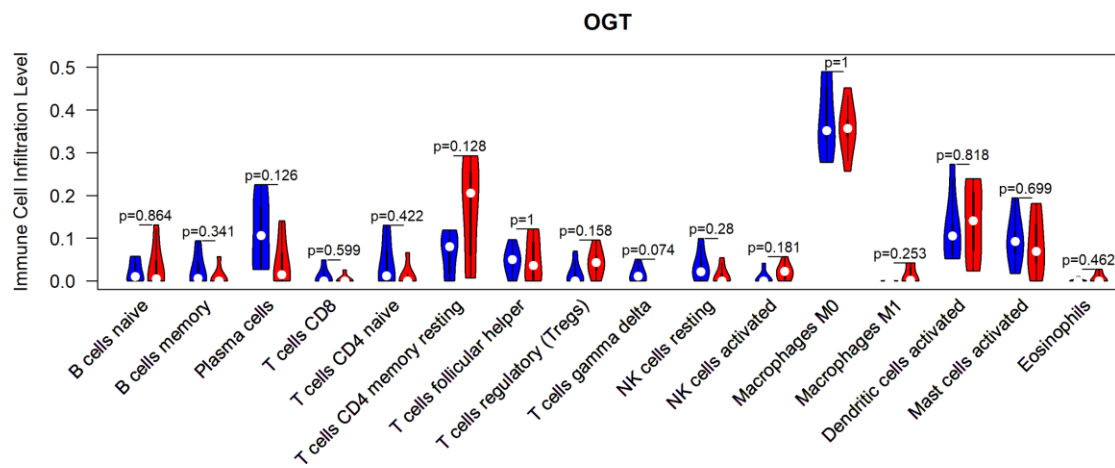

Supplement: Supplementary Materials — Table S1. Thirty-two cancers in the TCGA-GTEx cohort were eventually included in the study. Figure S1. The processes of selecting GEO cohorts. Figure S2. The frequency of SNVs of OGT in pan-cancer. Figure S3. SNVs of the high-OGT expression group and the low-OTG expression group. Figure S4. The relationship of OGT expression with clinical features in cancers. Figure S5. The prediction effect of OGT in pan-cancer. Figure S6. The eight datasets did not indicate that the expression of OGT was statistically different between the SCLC group and the nonSCLC group. Figure S7. No significant differences in clinical features were detected between the SCLC and nonSCLC groups. Figure S8. No statistical difference was detected in OGT expression and IME. [file 2010341.f1.zip › 2010341.f1/Figure S8.pdf]
